# The Dehgolan Prospective Cohort Study (DehPCS) on non-communicable diseases in a Kurdish community in the west of Iran

**DOI:** 10.4178/epih.e2021075

**Published:** 2021-10-01

**Authors:** Farhad Moradpour, Ebrahim Ghaderi, Ghobad Moradi, Mojdeh Zarei, Amjad Mohamadi Bolbanabad, Bakhtiar Piroozi, Azad Shokri

**Affiliations:** 1 Social Determinants of Health Research Center, Research Institute for Health Development, Kurdistan University of Medical Sciences, Sanandaj, Iran; 2 Department of Health in Emergencies and Disasters, School of Public Health, Tehran University of Medical Sciences, Tehran, Iran; 3 Deputy of Research and Technology, Kurdistan University of Medical Sciences, Sanandaj, Iran

**Keywords:** Cohort study, Non communicable disease, PERSIAN cohort, Risk factors

## Abstract

The Dehgolan Prospective Cohort Study (DehPCS) was conducted to examine and identify risk factors for the most prevalent non-communicable diseases (NCDs). In addition, in order to examine participants’ health status, socioeconomic status, behavioral factors, nutritional status, and environmental exposures, the DehPCS collected, analyzed, and stored blood, urine, nail, and hair samples to conduct genetic studies and identify biomarkers and other biological determinants of NCDs. In total, 3,996 adults aged 35 to 70 from the general population participated in the study from February 2018 to March 2019. Of them, 43.7% were women. The first follow-up wave was conducted with 3,995 participants. Information on a wide range of variables was collected, including on socioeconomic status, lifestyle, nutritional status, habits, physical examination findings, medication use, and medical history. Proxy variables such as body mass index, metabolic equivalent task score, wealth index, and macronutrients and micronutrients were calculated. The most common self-reported diseases in descending order were kidney stones, hypertension, and fatty liver. The prevalence of diabetes and hypertension was 9.3% and 33.4%, respectively. All data, samples, and measurements will be collected again at 5-year intervals. Thus, it will be possible to examine time-dependent changes in the risk factors of NCDs. The DehPCS can be used to study the relationships among genetics, lifestyle, socioeconomic status, and environmental risk factors and the most prevalent NCDs in case-cohort studies using a nested case-control design that will be applied to the cohort infrastructure. Researchers can also submit pre-proposals via the following web address: http://c.ddrc.ac.ir/persianaccess/Account/Login.

## INTRODUCTION

The epidemiological transition and demographic changes around the world have led to increased prioritization of non-communicable diseases (NCDs). Chronic diseases such as cardiovascular disease, diabetes, cancer, and chronic lung disease are now responsible for higher rates of morbidity and mortality than other causes of death. In recent years, low-income and middle-income countries have seen an increasing incidence of NCDs and deaths resulting from NCDs [1].

In Iran, which is considered a middle-income country, NCDs are responsible for approximately 83.48% (82.64-84.29) of all deaths nationwide and comprise 78.09% (76.05-80.05) of the nation’s disease burden. It has been estimated that, in 2019, 326,507 deaths occurred due to NCDs in Iran, and during the last 20 years, the number of deaths caused by NCDs has increased in Iran by 18.40%. In addition, the rate of premature death (death from the ages of 15 to 49) due to NCDs in Iran was found to be 65.28 deaths per 100,000 population (52.92%) [2].

Ischemic heart disease (IHD) is the leading cause of death in Iran, causing 26.28% of deaths nationwide, followed by cancer, stroke, diabetes, and kidney disease, which cause 17.08%, 10.46%, and 7.03% of deaths nationwide, respectively [3]. Studies have shown that more than 5 million Iranians suffer from diabetes, corresponding to an increase of about 113% compared to 2005. High blood pressure, high body mass index (BMI), high fasting blood sugar, smoking, and high levels of low-density lipoprotein cholesterol are important risk factors for NCDs, which comprise the first to fifth-ranked factors accounting for 42.12% of the disease burden [2]. The high number of cases and deaths due to NCDs in Iran has attracted the attention of politicians and is considered a priority by the government. Through effective health programs, mobilization of facilities, and inter- and intra-sector cooperation, a national action plan for the prevention and control of NCDs has also been implemented in Iran. However, an increase in the urban population, lifestyle changes, and an increase in the life expectancy in recent years, as well as the predicted rapid aging of the population in the coming years, have further complicated the situation [4,5].

Population-based prospective cohort studies are ideal for examining the effects of genetic susceptibility, occupation, environment, socioeconomic status, nutrition, and other lifestyle-related aspects on the occurrence of NCDs. For this purpose, to investigate the environmental effects and the effects of ethnicity and to identify major modifiable risk factors in different parts of Iran, the comprehensive and uniform Prospective Epidemiological Research in Iran (PERSIAN) initiative was launched in 2014 with the support of the Ministry of Health and Medical Education and the Digestive Diseases Research Institute [6]. Currently, 18 cohorts, including the DehPCS, operate in different parts of the country according to the same protocol. DehPCS and the Ravansar NonCommunicable Disease study [7] both examine public health in Kurdish communities; however, the physical environment and the environmental exposures of the inhabitants of these 2 regions are completely different.

The goals of the DehPCS are as follows: (1) to determine the incidence of major NCDs (cardiovascular disease, cerebrovascular disease, cancer, diabetes, chronic lung disease, chronic kidney disease, and liver disease) among the 35-year-old to 70-year-old Kurdish population in Dehgolan, Kurdistan Province, in the west of Iran; (2) to determine the trends in hospital admission and all cause-mortality/cause-specific mortality due to NCDs; (3) to evaluate trends over time in lifestyle, socioeconomic status, environmental factors, nutrition, and the outcomes of interest in the Kurdish population; (4) to compare and clarify the relationship between risk factors (e.g., pesticides, opioid use, nutrition, dietary patterns, economic inequality) and NCDs; (5) to establish a biobank for basic genetic studies and to discover biomarkers and other biological determinants of NCDs; and (6) to establish a population-based structure and cooperate with other PERSIAN cohort centers to increase the strength of the findings from the cohort data set.

**Figure 1.**
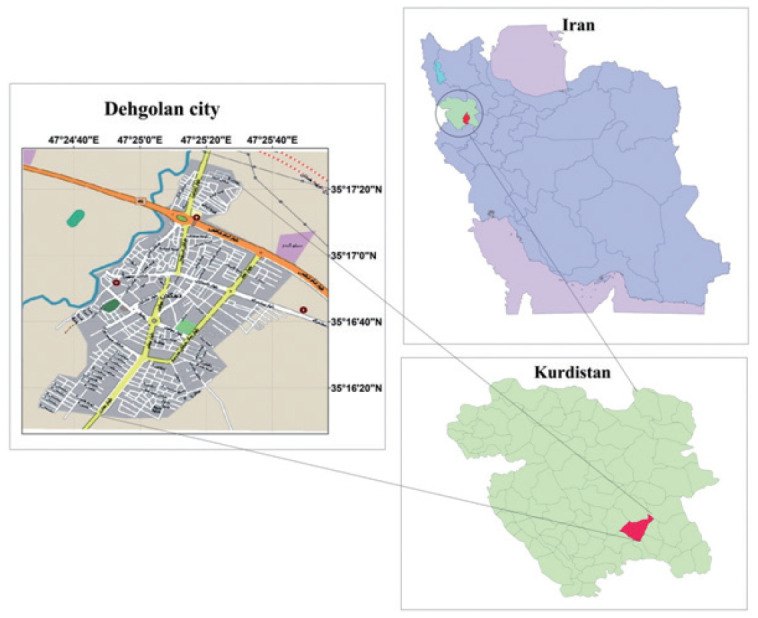
Location of Kurdistan Province and the Dehgolan district in Iran.

DehPCS was designed to examine NCDs among the Kurdish population. Geographically, the Kurdish people are located across a wide area between the northwest of the Zagros Mountains and the eastern Taurus Mountains, which includes the east and southeast of Turkey, the north and northeast of Iraq, the west and northwest of Iran, and the north of Syria and has an approximate area of 392,000 km^2^ (Figure 1) [6]. The Kurdish population is estimated at 41 million people, and 10 million to 12 million Kurds are estimated to live in Iran [8]. The DehPCS was conducted in Dehgolan city in the south of Kurdistan Province. Kurdistan Province, with an area of 29,137 km^2^, has a population of about 1,600,000. The capital of the province is Sanandaj, which has a population of approximately 580,000 and is located 45 km west of Dehgolan. Dehgolan County, at the center of which lies the city of Dehgolan, has a population of about 68,000. The city of Dehgolan has a population of about 26,000, almost all of which is composed of Kurds. Dehgolan County contains 1 hospital, 2 comprehensive urban health service centers, 4 comprehensive rural service centers, and 41 health houses [9].

The city of Dehgolan was selected for this study for the following reasons: Dehgolan is located 45 km from Kurdistan University of Medical Sciences and was easily accessible to the researchers on the Sanandaj-Tehran communication route; The majority of the Dehgolan population is Kurdish and they are similar to the Kurds of other regions in terms of culture, food, and lifestyle; Due to low migration in Dehgolan, dropout from the study in the follow-up phase will be minimized; Easy access to the population and familiarity of the study staff with the population reduce costs and increase the response rate.

## STUDY PARTICIPANTS

The enrollment phase commenced in February 2019. The pilot phase of the study lasted until May 2019, during which 984 people enrolled. The participants in this study included only individuals who resided in the urban area of Dehgolan. Inclusion criteria for participants included: (1) 35 years to 70 years of age; (2) Iranian citizenship (based on possession of a national identification card and birth certificate); (3) a residence history of at least 1 year with a duration of at least 9 months per year; and (4) eagerness to participate in the study and provide informed consent, and having the necessary ability to communicate with the team of interviewers. Blind, deaf, and mute people as well as individuals with mental disorders (such as untreated psychosis) who were unable to participate in the questioning process were excluded from the study. The age range of 35 years to 70 years old (adults) was picked for 3 reasons. First, people in this age group are more likely to have well-established lifestyles and behaviors. Second, those in this age group were more likely to reach the study outcome of interest within a reasonable time. Third, people in this age group are more likely to be active and energetic enough to participate in the study.

To select the sample, the study team conducted a dedicated census. Systematic random 1-stage cluster sampling was used to select the participants. We decided to include 50% of all 721 eligible blocks in the study, and the sampling ratio was thus set to 2. The first block was chosen by randomly selecting a value equal to or less than the sampling ratio. The next blocks were selected by adding the row number of the previous block to the sampling ratio. All eligible individuals were asked to participate in the study. Sampling started from the first building on the right side of the closest street to the cohort study center. A local, trained interviewer who spoke Kurdish conducted the sample selection by visiting urban area households on a door-to-door basis, conducting a census, registering the addresses of households, and assigning a special code to each household. Information of household members, including their names, ages, genders, positions in the family, and contact numbers, was collected. The goals of the study and its implementation process were discussed with family members, and their questions were answered. When at least 1 eligible family member agreed to enroll in the study, a pamphlet was provided that contained information about the purpose of the study, its implementation methods, the required tests, and the required conditions for the laboratory tests (such as fasting and tests performed on blood and urine samples). In addition, after individuals accepted the invitation to participate, they were assigned a specific time to visit the cohort study center. Individuals who did not appear at the appointed time were not immediately excluded from the study and were only excluded if they were absent after 3 calls or after setting a new appointment.

A reminder phone call was made the day before a participant’s arrival. One week before their assigned time to visit the cohort study center, participants were advised not to cut their nails or dye their hair and to fast for at least 8 hours.

Approximately 9,000 people aged 35 to 70 live in the Dehgolan area. Based on the available resources and in coordination with the central team, the members of the research team decided to include 4,000 people in the study. All eligible individuals were invited to enroll in the study. Individuals’ reasons for refusing to participate in the study for those who did not wish to enroll or who failed to appear at their appointment at the cohort study center were recorded. Of those who were invited, 404 (9.2%) refused to participate in the study for the following reasons: (1) lack of sufficient time or being busy with work (41.0%); (2) lack of confidence in the study (17.0%); (3) rumors of acquired immune deficiency syndrome transmission and an increased risk of other diseases such as influenza and coronavirus disease 2019 (14.0%); (4) caretaking duties for a child or elderly person at home (9.0%); (5) fear of sampling and testing (6.0%); and (6) other reasons, such as travel, family disapproval, or forgetting the attendance date (12.0%).

**Figure 2.**
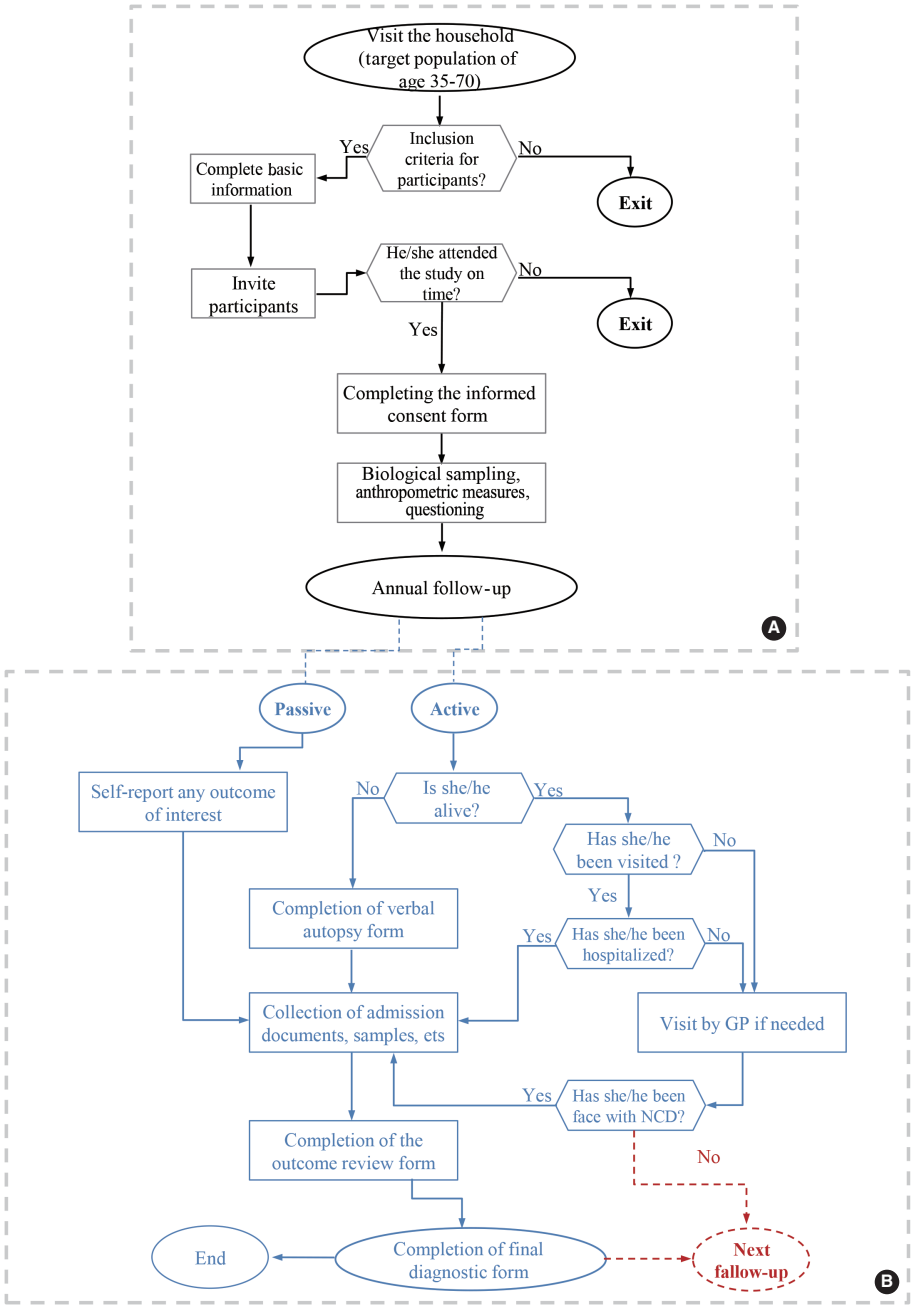
Algorithm of the baseline measurements of Dehgolan Prospective Cohort Study (DehPCS), 2018-2019 (A) and assessing individuals’ conditions and medical events during the annual follow-up of DehPCS (B). NCD, non-communicable diseases; GP, general practitioner.

When attending the cohort center, the eligibility criteria of the participants were approved by the trained interviewer. The participants completed informed consent forms, and the identifying characteristics of participants and their ages were registered online based on their national and identity cards. Each participant was given a unique, 11-digit identification code. After registration, the participants were referred to a laboratory for blood, urine, hair, and nail samples (Figure 2A).

### Ethics statement

The design and implementation of the study were approved by the Ethics Committee of Kurdistan University of Medical Sciences and was assigned the ethical approval code IR.MUK.REC. 1396/93.

## MEASUREMENTS

### Follow-up

The first wave of follow-up commenced 1 year after enrollment in February 2020. Follow-up with participants is conducted on an annual basis, both actively (by phone) and passively (self-reports and reports from cancer and disease registries, private physicians, forensic physicians, insurance institutes, and laboratories). In most cases, follow-up is conducted over the phone by the study’s interviewer and physician at 1-year intervals or when an outcome of interest occurs. If the person or his/her relatives do not answer the phone after 6 attempts over 2 weeks (3 different days each week), the executive follow-up team first refers to the person’s postal address, and if the person is not present at the identified address, they refer to his/her relatives’ postal addresses to conduct a face-to-face follow-up. In addition, if necessary and according to the fieldwork schedule, a face-to-face interview can be conducted in the field to complete the follow-up after re-inviting participants (Figure 2B).

Outcome review forms (for chronic NCDs) and verbal autopsies (in case of death) are completed by the physician to obtain the desired outcomes. Living participants are invited to visit the cohort site for further examination. To reduce recall bias, participants are asked to provide hospitalization information and documentation related to diagnostic and treatment procedures and medications. In addition, clinical, laboratory, and disease profile information for each case (the occurrence of outcomes) in all hospitals and laboratories are collected by an inquiring physician, and a copy of the corresponding profile is prepared. The information recorded on these forms, along with all previous follow-up questionnaires, laboratory test results, existing biological samples, death certificate images, and other necessary medical records, is reviewed for the final diagnosis by members of the outcome review committee, which is composed of 2 physicians (internal medicine specialists) who complete the final diagnosis form based on the evidence, depending on the outcome type or cause of death. If the final diagnosis of the 2 physicians does not match, a third reviewer (an internal medicine specialist or another specialist) will be consulted.

To investigate changes in risk factors or protective factors, all data, samples, and measurements from the registration phase will be collected again in the 5th, 10th, and 15th years. Blood samples will also be collected as soon as possible for participants in whom an outcome of interest occurs.

Participation in the study ends if participants are unwilling to continue with the study, move or are otherwise unable to be reached, are unable to recall information or provide information that is unreliable, or die. If a participant is unwilling to continue with the study, the executive team during follow-up will try to re-invite the participant to the study by outlining the benefits of participation, including improvement of individual and community health outcomes, access to mostly free medical services at study centers for early diagnosis (and treatment) of cancer and other diseases, and benefits as a result of recommendations for the prevention of chronic diseases, especially heart attacks and strokes. If a participant moves or is unable to be reached during follow-up, the executive team during follow-up will try to re-contact the participant according to the aforementioned identity information and contact methods.

As of this paper, the first wave of the follow-up phase has been completed. The response rate of the first wave of follow-up was 3,995 participants (99.97%). Only 1 person was unwilling to continue the study. The second wave of follow-up has commenced as of the writing of this paper, but it has not yet been completed.

### Instruments and measurements

Upon arrival, all participants were initially enrolled using online software. Unique 11-digit codes referred to as the PERSIAN code identification (PCID) were assigned to them by the software. The participants were then immediately referred to biological sampling since they had fasted up to that point.

Laboratory evaluation: A 25 mL blood sample was taken using a standard method (sitting and using a tourniquet) with vacuum tubes. In addition, 15 mL of urine was collected in sterile containers. Approximately 300 strands of hair with a width of up to 3 cm were taken from the back of the head, and at least 10 nail samples were taken from the participants. Blood samples were divided into different aliquots using a 3,000 rpm centrifuge for 10-15 minutes (Table 1). After labeling the samples with the participants’ 11-digit PCID codes, the aliquots were placed in a freezer at -70°C. In addition, small amounts of blood samples were used for the following tests: complete blood count, blood lipid profile, blood glucose profile, alkaline phosphate, creatinine, blood urea nitrogen, gamma glutamine transpeptidase, alanine transaminase, and aspartate transaminase. Urine samples were analyzed for urine pH, blood, glucose, bilirubin, nitrate, ketones, ascorbic acid, leukocytes, microalbuminuria, and specific gravity. The results of all analyses were stored in a database and provided to the participants as an incentive to continue with the study. The samples were stored for 24 hours a day using storage equipment with automatic monitoring sensors and an uninterruptible power supply.

#### Anthropometry

To standardize anthropometry and reduce the systematic error, anthropometric measurements were performed after sample collection when participants were still fasting. Participants were asked to remove their shoes, heavy clothes, and accessories. A Seka standing hand scale and Seka inelastic tape measure with an accuracy of 0.1 cm were used for taking measurements. The waist circumference, hip circumference, wrist circumference, height (cm) and weight (kg) were measured according to the National Health Protocol [10].

#### Questioning

A wide range of questions were answered by participants in the DehPCS. Trained interviewers asked each participant a total of 536 questions across 3 areas (general, medicine, and nutrition) which were answered on a face-to-face basis. Whenever possible, standard validated questionnaires for the Iranian population were used to collect data [11-13].

General questions covered general and demographic characteristics (42 questions), socioeconomic status (27 questions), work history (7 questions), lifestyle (36 questions), sleep and physical activity (48 questions), and personal habits (14 questions). In addition, the level of participants’ trust in the health care system was measured using 2 questions (Table 2). Data from this section were collected after measuring the anthropometric characteristics of the participants.

Questions related to medical status covered fertility history (33 questions), medical history (81 questions), medications (3 questions), family history of diseases (27 questions), dental and oral health (9 questions), physical examinations and blood pressure (25 questions), and general health (29 questions) (Table 2).

The nutritional status of participants was assessed using 3 questionnaires covering food frequency (112 questions), dietary supplements (10 questions), and eating habits (31 questions). The food frequency questionnaire is a tool that examines respondents’ consumption of food and beverages in the past year. The questionnaire included items specific to the Iranian population such as bread, cereals, meats, legumes, dairy products, fruits and vegetables, sugars and sweets, and a variety of oils. Local foods and breads were also added to the questionnaire. In this section, questions pertaining to cooking methods and food storage were also asked (Table 2).

During the annual follow-up, participants’ contact information (e.g., the address and telephone number of the participant and his/her relatives) is updated. All medical records from the previous year, including vital signs, hospital admissions, medical events, diagnostic and therapeutic measures, medication regimens, and final results, are also registered.

## KEY FINDINGS

A total of 3,985 (99.7%) of the 3,996 participants in the DehPCS were of the Kurdish ethnicity. Men comprised 1,748 (43.7%) of the sample, and 1,786 (44.7%) were middle-aged adults, while only 486 (12.2%) were more than 60 years old (elderly). In total, 3,671 (91.9%) were married and 44 (1.1%) were single (never married). Of the participants, 1,247 (31.2%) were illiterate and only 514 (12.9%) had a university degree. The number of participants who used the internet was 1,113 (28.1%). Socioeconomic status based on a wealth index showed that 811 (20.4%) participants were classified as belonging to the poorest quintile, while 663 (16.7%) were in the wealthiest quintile (Table 3).

Overall, 2,991 (75.1%) participants—particularly women— were overweight or obese based on BMI, and only 944 (23.7%) participants were normal weight. According to the WHO criteria regarding waist-to-hip ratio (WHR), 575 (14.4%) of the participants, most of whom were women, had a WHR of more than 1 and were thus at a higher risk for heart disease. In total, only 547 (13.8%) participants, most of whom were men, had a physical activity level equal to or higher than 2,700 metabolic equivalent task-minutes per 24 hours. Lastly, 1,020 (25.6%) of the participants consumed equal to or more than the upper tolerable level of 5 grams of salt per day (Table 4).

The most prevalent chronic diseases in descending order were kidney stones, hypertension, fatty liver, psychiatric disorders, chronic headaches, thyroid diseases, and diabetes. According to the medication intake of participants as well as their fasting plasma glucose and blood pressure measurements, hypertension was present in 1,334 participants (33.4% men, 32.6% women, and 34.0% overall), and diabetes was found in 373 participants (9.3% men, 7.9% women, and 10.5% overall). Apart from kidney stones, which are a different type of disease, most NCDs were more common among women than among men (Table 5).

## STRENGTHS AND WEAKNESSES

The DehPCS, as a part of the larger PERSIAN cohort study, makes it possible to investigate the relationship between geographical environment and ethnicity on the occurrence of NCDs. In addition, this study as well as a study of NCDs in the Ravansar cohort are among the first prospective cohort studies to examine the major risk factors for NCDs among the Kurdish population as an independent ethnic group. It should be noted that the rate of participation in this study was high.

In this study, some baseline variables were collected on a self-reported basis by participants. Thus, the study might have limitations due to information bias, as in other similar studies. In addition, people who elect to participate in long-term studies such as cohort studies tend to differ from other members of the community in some respects. For example, participants in such studies may tend to be more concerned about their health than others, which could limit the study in terms of selection bias.

## DATA ACCESSIBILITY

Although the data from this study are not freely accessible, in order to increase scientific output, international researchers can access the study data for secondary analysis by submitting a request under the terms and conditions of the publication of PERSIAN cohort data. All necessary information about how to access the data, such as authorship rules, data transfer agreements and samples, and the data dictionary, is available on our website at https://persiancohort.com. Researchers can also submit their pre-proposals via the following web address: http://c.ddrc.ac.ir/persianaccess/Account/Login.

## Figures and Tables

**Table 1.** Characteristics of biological samples stored in the DehPCS biobank

Sample	No. of aliquots (sample amount per 1 mL)	Storage conditions (°C)
Blood		
Whole blood	4	-70
Plasma	6	-70
Buffy coat	2	-70
Serum	3	-70
Urine	1	-20
Hair	About 300 strands of hair for a size of 1-3 cm	In foil and zip-up bag with silica gel in cool and dry storage
Nails	10 fingernails and toenails	In foil and zip-up bag with silica gel in cool and dry storage

DehPCS, Dehgolan Prospective Cohort Study.

**Table 2.** Measures and instruments used in baseline data collection and 5-year follow-up assessments for the DehPCS

Categories	Groups	Measures & Instruments
General	General & demographic characteristics	Birth date, birthplace, gender, age, ethnicity, contact address, marriage status, type and no. of marriage (first, second degree), participant's status in the household, family size, no. of bedrooms
	Socioeconomic characteristics	Education, employment status, assets, job history, status of current residence, area of residence, wealth score index, source of income, dwelling status, annual reading status
	Environmental exposure	Drinking water status, use of a kitchen hood or ventilator, contact with animals, fuel use for cooking and heating, access to a landline, cell phone access, internet access, trip status, hygiene status and the status of hygienic facilities, housing type/structure, exposure to agriculture/household pesticides
	Lifestyle	Circadian rhythm of food and sleep, physical activity, sleep quality, shift work, daily activity
	Personal habits	History of smoking and passive smoking, alcohol consumption, drug use
Medical	Reproductive history	Menstrual cycle, history of live birth, abortion, stillbirth, history of breastfeeding, history of infertility, oophorectomy, tubectomy, hysterectomy, history of contraceptive use, history of hormone replacement therapy, history of breast exams, mammography, and Pap smear
	General health & medical history	General health status including physical symptoms, anxiety, social function and depression, subjective health status, family history of illness, diabetes and renal disease, cardiovascular disease, liver disease, chronic lung disease, thyroid disease, kidney disease, cancer, psychological disorders, neurological disease, other sign and symptoms, history of transfusion, hospitalization history, surgery, disability and amputation
	Medication use	Type, time frame, and duration of medication use
	Dental health	History of brushing and flossing, no. of teeth, decayed teeth, missing teeth, teeth with fillings, oral lesions
	Blood pressure & physical exam anthropometry	Right and left blood pressure, heart rate, alopecia, hirsutism, iris color, physical or sensational disabilities, spinal disorders
		Height, weight, hip circumference, waist circumference, wrist circumference, body mass index Complete blood count, liver function tests, renal function tests, lipid profile, fasting blood sugar level
	Blood & urine analysis	Complete blood count, liver function tests, renal function tests, lipid profile, fasting blood sugar level
Nutrition	Food frequency	125-item food frequency questionnaire on the diet of participants in recent years, covering bread and cereals, beans, meat and meat products, dairy products, fruits and vegetables, oils and oilseeds, sugars, and spices
	Supplement use	Type and amount of supplements used in the past year
	Dietary habits	Cooking method, food preparation and preservation, dietary habits in the past year, food allergy status

DehPCS, Dehgolan Prospective Cohort Study.

**Table 3.** Demographic characteristics and socioeconomic status of DehPCS participants

Variables	Total	Men	Women	p-value (χ^2^)
Age (yr)				
35-45	1,786 (44.7)	733 (41.9)	1,053 (46.8)	0.005
46-60	1,724 (43.0)	782 (44.8)	942 (41.9)	
61-70	486 (12.7)	233 (13.3)	253 (11.3)	
Marital status				
Single	44 (1.1)	4 (0.2)	40 (1.8)	<0.001
Married	3,671 (91.9)	1,729 (86.4)	1,942 (98.9)	
Widowed	252 (6.3)	243 (10.8)	9 (0.5)	
Divorced	28 (0.7)	5 (0.3)	23 (1.0)	
Education level (yr)				
Illiterate	1,247 (31.2)	185 (10.9)	1,062 (47.2)	<0.001
1-5	1,112 (27.8)	458 (26.2)	654 (29.1)	
6-12	1,123 (28.1)	691 (39.5)	432 (19.2)	
University	514 (12.9)	414 (23.7)	100 (4.5)	
Consanguineous marriage				
Yes	809 (20.5)	363 (20.8)	446 (20.2)	0.630
No	3,143 (79.5)	1,381 (79.2)	1,762 (79.8)	
Mobile phone access				
Yes	3,632 (91.7)	1,712 (99.1)	1,920 (85.9)	<0.001
No	330 (8.3)	15 (0.9)	315 (14.1)	
Internet access				
Yes	1,113 (28.1)	671 (38.9)	442 (19.7)	<0.001
No	2,849 (71.9)	1,056 (61.1)	1,793 (80.3)	
Wealth index				
Q1 (poorest)	811 (20.4)	-	-	-
Q2	824 (20.8)	-	-	
Q3	933 (25.0)	-	-	
Q4	679 (17.1)	-	-	
Q5 (wealthiest)	663 (16.7)	-	-	

Values are presented as number (%). DehPCS, Dehgolan Prospective Cohort Study.

**Table 4.** Anthropometric indices, nutrition, and substance use among DehPCS participants

Variables	Total	Men	Women	p-value (χ^2^)
BMI (kg/m^2^)				
Underweight (<18.5)	45 (1.1)	30 (1.7)	15 (0.7)	<0.001
Normal or healthy weight (18.5-24.9)	944 (23.7)	608 (34.9)	336 (15.0)	
Overweight (25.0-29.9)	1,705 (42.8)	774 (44.5)	931 (41.6)	
Obese (≥30.0)	1,286 (32.3)	329 (18.9)	957 (42.7)	
WHR (WHO)^1^				
Healthy	1,229 (30.9)	574 (33.0)	655 (29.3)	<0.001
Overweight	2,176 (54.7)	998 (57.3)	1,178 (52.6)	
At risk for heart disease	575 (14.4)	169 (9.7)	406 (18.1)	
Smoking				
Yes	927 (23.5)	787 (45.7)	140 (6.3)	<0.001
No	3,024 (76.5)	636 (54.3)	2,088 (93.7)	
Alcohol consumption				
Yes	481 (12.2)	437 (25.4)	44 (2.0)	<0.001
No	3,470 (87.8)	1,286 (74.6)	2,184 (98.0)	
Drug use				
Yes	448 (11.3)	424 (24.6)	24 (1.1)	<0.001
No	3,502 (88.7)	1,298 (75.4)	2,204 (98.9)	
MET-min (daily)				
1,440-2,100	1,100 (27.8)	610 (35.4)	490 (21.9)	<0.001
2,101-2,699	2,312 (58.4)	636 (36.8)	1,676 (75.1)	
≥2,700	547 (13.8)	480 (28.7)	67 (3.0)	
Duration of sleep (hr/d)				
<7	1,584 (40.0)	743 (43.0)	841 (37.7)	0.001
≥7	2,376 (60.0)	985 (57.0)	1,391 (62.3)	
Fried food intake (meals/mo)				
<4	961 (24.2)	381 (21.9)	580 (25.9)	0.009
4-12	2,834 (71.2)	1,267 (72.9)	1,567 (69.9)	
>12	185 (4.6)	90 (5.2)	95 (4.2)	
Dyslipidemia				
Yes	3,228 (80.8)	1,301 (74.4)	1,927 (85.7)	<0.001
No	768 (19.2)	447 (25.6)	321 (14.3)	
Salt intake (g/d)				
<5	2,958 (74.4)	1,253 (72.1)	1,705 (76.1)	0.005
≥5	1,020 (25.6)	484 (27.9)	536 (23.9)	

Values are presented as number (%). DehPCS, Dehgolan Prospective Cohort Study; BMI, body mass index; MET, metabolic equivalent task; WHO, World Health Organization; WHR, waist-to-hip ratio.

1 Healthy: less than 0.80 and 0.90 for women and men respectively; Overweight: between 0.8-1.0 for women and 0.9-1.0 for men; At risk for heart disease: higher than 1 in women and men.

**Table 5.** Prevalence of chronic and underlying diseases diagnosed by a physician based on the self-reports of DehPCS participants

Variables	Total	Men	Women	p-value (χ^2^)
Diabetes				
Yes	522 (13.2)	151 (9.0)	371 (16.6)	<0.001
No	3,440 (86.8)	1,576 (91.0)	1,864 (83.4)	
Hypertension				
Yes	774 (19.5)	226 (13.1)	548 (24.5)	<0.001
No	3,188 (80.5)	1,501 (86.9)	1,687 (75.5)	
Ischemic heart disease				
Yes	300 (7.6)	118 (6.8)	182 (8.1)	0.068
No	3,662 (92.4)	1,609 (93.2)	2,053 (91.9)	
Stroke				
Yes	62 (1.6)	27 (1.6)	35 (1.6)	0.990
No	3,914 (98.4)	1,708 (98.4)	2,206 (98.4)	
Myocardial infarction				
Yes	73 (1.8)	48 (2.7)	25 (1.1)	<0.001
No	3,889 (98.2)	1,679 (97.3)	2,210 (98.9)	
Chronic lung diseases^1^				
Yes	163 (4.1)	46 (2.7)	117 (5.2)	<0.001
No	3,799 (95.9)	1,681 (97.3)	2,118 (94.8)	
Thyroid diseases^2^				
Yes	535 (13.5)	64 (3.7)	471 (21.1)	<0.001
No	3,427 (86.5)	1,663 (96.3)	1,764 (78.9)	
Fatty liver				
Yes	762 (19.2)	269 (15.5)	493 (22.0)	<0.001
No	3,214 (80.8)	1,466 (84.5)	1,748 (78.0)	
Kidney stones				
Yes	813 (20.5)	440 (25.4)	373 (16.6)	<0.001
No	3,163 (79.5)	1,295 (74.6)	1,868 (83.4)	
Chronic headaches				
Yes	608 (15.3)	99 (5.7)	509 (22.7)	<0.001
No	3,368 (84.7)	1,636 (94.3)	1,732 (77.3)	
Psychiatric disorders^3^				
Yes	672 (16.9)	257 (14.8)	415 (18.5)	0.002
No	3,304 (83.1)	1,478 (85.2)	1,826 (81.5)	

Values are presented as number (%). DehPCS, Dehgolan Prospective Cohort Study.

1 Including tuberculosis, asthma, etc.

2 Including hypothyroidism, hyperthyroidism, goiter, and thyroid nodules.

3 Diagnosed with any kind of psychiatric disorder other than depression.
